# Prevalence of psychoactive substance use in men who have sexual relationships with men, Colombia

**DOI:** 10.15649/cuidarte.3477

**Published:** 2024-07-26

**Authors:** Sebastián Bedoya Mejia, Doris Cardona Arango, Maite Catalina Agudelo Cifuentes, Sara Milena Ramos-Jaraba, Giselly Matagira-Rondón, Ángela M Segura-Cardona, Dedsy Yajaira Berbesí-Fernández

**Affiliations:** 1 Professor, PhD student in Epidemiology and Biostatistics, CES University, Medellín, Colombia. E-mail: sebedoya@ces.edu.co Universidad CES CES University Medellín Colombia sebedoya@ces.edu.co; 2 Professor, PhD in demographic, Medellín, Colombia. E-mail: doris.cardona@gmail.com Medellín Colombia doris.cardona@gmail.com; 3 Professor, PhD in Epidemiology and Biostatistics, Faculty of Nursing, CES University, Medellín, Colombia. E-mail: magudeloc@ces.edu.co Universidad CES Faculty of Nursing CES University Medellín Colombia magudeloc@ces.edu.co; 4 Professor, Master in Collective Health, Faculty of Nursing, CES University, Medellín, Colombia. E-mail: sramosj@ces.edu.co Universidad CES Faculty of Nursing CES University Medellín Colombia sramosj@ces.edu.co; 5 Professor, master’s in public health, Faculty of Nursing, CES University, Medellín, Colombia. E-mail: gmatagira@ces.edu.co Universidad CES Faculty of Nursing CES University Medellín Colombia gmatagira@ces.edu.co; 6 Professor, PhD in Epidemiology, Graduate School, CES University, Medellín, Colombia. E-mail: asegura@ces.edu.co Universidad CES CES University Medellín Colombia asegura@ces.edu.co; 7 Professor, PhD in Epidemiology and Biostatistics, Faculty of Nursing, CES University, Medellín, Colombia. E-mail: dberbesi@ces.edu.co Universidad CES Faculty of Nursing CES University Medellín Colombia dberbesi@ces.edu.co

**Keywords:** Sexual Behavior, Social Discrimination, Substance-Related Disorders, Sex Work, Comportamiento Sexual, Discriminación Social, Trastornos Relacionados con Sustancias, Trabajo Sexual, Comportamento Sexual, Discriminagáo Social, Transtornos Relacionados ao Uso de Substancias, Trabalho Sexual

## Abstract

**Introduction:**

The use of psychoactive substances (PS) in the population is a current problem that affects a large part of humanity, with diverse consequences.

**Objective:**

To analyze the factors associated with the consumption of PS among men who have sex with other men (MSM) in three Colombian cities.

**Material and Methods:**

This cross-sectional descriptive study used the respondent-driven sampling (RDS) method and obtained a sample of 1301 MSM. The association between the sociodemographic and personal characteristics and the consumption of PS was assessed using the chi-square test. Prevalence ratios were calculated along with their 95% confidence intervals. For the multivariate analysis, a Poisson regression with a log link and robust estimator was employed to explore the factors associated with PS use.

**Results:**

The prevalence of consumption of PS in the last year was 87%, prevailing the consumption of alcohol, marijuana, and poppers. Having occasional partners (PR: 0.44; 95% CI 0.29 - 0.67), attending public places or establishments such as bars and saunas (PR: 3.39; 95% CI 2.34 **-** 4.91), sex work, and not using a condom in the last sexual encounter (PR: 2.10 95% CI 1.37 - 3.22) are factors associated with the use of these substances.

**Discussion:**

There is evidence of a high prevalence of recreational use of PS, even higher than that found in the general population.

**Conclusion:**

A high prevalence and association with risky sexual behaviors is confirmed, which requires promotion and prevention actions to reduce the use of these substances.

## Resumen

**Introducción:**

El uso de sustancias psicoactivas en la población es un problema actual, que afecta a una gran parte de la humanidad, con diversas consecuencias.

**Objetivo:**

Analizar los factores asociados al consumo de sustancias psicoactivas en hombres que tienen sexo con otros hombres (HSH) en tres ciudades de Colombia.

**Materiales y Métodos:**

Este estudio descriptivo transversal utilizó el método de muestreo respondent-driven sampling y logró una muestra de 1301 HSH. Se evaluó la asociación entre las características sociodemográficas y personales y el consumo de sustancias psicoactivas mediante la prueba chi-cuadrado. Las razones de prevalencia se calcularon con sus intervalos de confianza del 95%. En el análisis multivariado, se empleó una regresión de Poisson con enlace logarítmico y estimador robusto, para explorar los factores asociados con el uso de sustancias psicoactivas.

**Resultados:**

La prevalencia de consumo de sustancias psicoactivas en el último año fue del 87%, predominando el consumo de alcohol, marihuana y poppers. Tener parejas ocasionales (RP: 0,44; IC 95% 0,29-0,67), asistir a lugares públicos o establecimientos como bares y saunas (RP: 3,39; IC 95% 2,34 - 4,91), el trabajo sexual y no usar condón en la última relación sexual (RP: 2,10; IC 95%: 1,37 - 3,22) son factores asociados con el consumo de estas sustancias.

**Discusión:**

Se evidencia una alta prevalencia de consumo recreativo de sustancias psicoactivas, incluso superior a la encontrada en población general.

**Conclusión:**

Se confirma una alta prevalencia y asociación con conductas sexuales de riesgo, lo que requiere acciones de promoción y prevención para reducir el uso de estas sustancias.

## Resumo

**Introduçâo:**

O uso de substâncias psicoativas pela populaçâo é um problema atual que atinge grande parte da humanidade, com diversas consequências.

**Objetivo:**

Analisar os fatores associados ao uso de substâncias psicoativas em homens que fazem sexo com homens (HSH) em três cidades da Colômbia.

**Materiais e Métodos:**

Esse estudo descritivo transversal usou o método de amostras respondent-driven sampling e obteve uma amostra de 1301 HSH. A associaçâo entre as características sociodemográficas e pessoais e o uso de substâncias psicoativas foi avaliada por meio do teste do qui-quadrado. As razóes de prevalência foram obtidas com seus intervalos de confiança de 95%. Na análise multivariada, foram construidos regressâo de Poisson, log link e estimador robusto para explorar os fatores associados ao uso de substâncias psicoativas.

**Resultados:**

A prevalência de consumo de substâncias psicoativas no último ano foi de 87%, prevalecendo o consumo de álcool, maconha e poppers. Ter parceiros ocasionais (RP: 0,44; IC 95% 0,29 - 0,67), frequentar locais e locais públicos como bares e saunas (RP: 3,39; IC 95% 2,34-4,91), trabalho sexual e nao usar camisinha na última relaçâo sexual (RP: 2,10 IC 95% 1,37-3,22) sao fatores associados ao consumo dessas substâncias.

**Discussâo:**

Existem evidências de elevada prevalência de uso recreativo de substâncias psicoativas, ainda maior do que as encontradas na populaçao em geral.

**Conclusâo:**

Confirma-se uma elevada prevalência e associaçâo com comportamentos sexuais de risco, o que requer açôes de promoçâo e prevençâo para reduzir o uso destas substâncias.

## Introduction

The consumption of psychoactive substances (PS) is a phenomenon of interest for public health; it is estimated that 5.50% ofthe world's population in 2017 had used drugs and that more than 500,000 people lost their lives that year for that reason^1^. The excessive and systematic increase in the use of cocaine, amphetamines, ecstasy, and other synthetic and semi-synthetic opioids has led to chronic harmful effects on population health, alongside multiple social consequences. Additionally, the high consumption of alcohol and tobacco further exacerbates these problems^2^^, ^^3^.

Substance use increases the risk of sexually transmitted infections, such as the human immunodeficiency virus and the acquired immunodeficiency syndrome (HIV/AIDS), and hepatitis B and C, as well as chronic diseases like cancer, cardiovascular problems, and cirrhosis. It also affects mental health, leading to increased rates of suicide attempts, depression, anxiety, panic attacks, helplessness, paranoia, hallucinations, violent behavior, and psychosis. Socially, substance use can cause radical changes in performance, frequent absences from work or classes, changes in attitude within family and social environments, and sudden ‘personality’ changes. Legally, substance use is associated with criminal behaviors such as robbery, violence, drug trafficking, and illicit enrichment^4^.

Men who have sex with men (MSM) represent one of the groups with the highest consumption of PS. This heterogeneous group includes all men (biological males at birth) who have sex with other men, whether occasionally or frequently and encompasses straight, bisexual, and gay men, among others. Some studies indicate that MSM experience mental health issues and high rates ofdisorders, including depression^5^, suicidal tendencies, personality disorders, internalized homonegativity, and the use of legal and illegal substances.

Recreational drug use among MSM is not a recent phenomenon. Drugs have traditionally been used in nightlife and entertainment venues for behavioral disinhibition. While this reality is also observed in the heterosexual population, its prevalence has increased over the last twenty years across different countries, with variations depending on the substances available in each region. The consequences of this practice have raised alarms globally, as MSM who use drugs during sex are more likely to engage in risky behaviors, increasing the risk of acquiring diseases such as HIV^6^^)^ and experiencing rectal trauma from ‘sex marathons,’ which facilitate the transmission of various sexually transmitted infections (STIs). Additionally, HIV-positive MSM may be less adherent to antiretroviral therapy (ART) during chemsex, and the combined use of various medications can lead to drug dependence, acute intoxication, and even death^7^^, ^^9^.

Chemsex, a term combining the words ‘chemicals’ and ‘sex,’ refers to the use of stimulant substances associated with sexual activity within the MSM population. Typically, it involves the use of psychoactive drugs such as mephedrone, gamma hydroxybutyrate (GHB), gamma-butyrolactone (GBL), and crystallized methamphetamine before or during sexual activity^7^. These substances are often used in combination to facilitate prolonged sex sessions lasting several hours or days with multiple sexual partners. They are physiological stimulants that increase heart rate and blood pressure and trigger euphoria and sexual arousal^9^. Some users report using them to control negative feelings, such as a lack of confidence and self-esteem. A study conducted in Spain revealed alarming results: 60.00% of HIV-positive MSM and 63.30% of HIV-negative MSM were diagnosed with various STIs after engaging in chemsex sessions. The most common STIs include HIV, syphilis, gonorrhea, human papillomavirus (HPV), chlamydia, and hepatitis C^10^. Other prevalent practices in this population are barebacking (engaging in sexual activity without using a condom) and the use of unprotected sex toys.

Numerous studies have investigated the use of PS among MSM. For instance, a study in Spain revealed that more than 55.00% of MSM participants had used recreational drugs in the past twelve months, and 80.00% had done so to have sex^7^. In Colombia, seroprevalence studies from 2015 found similar results: over 90.00% of MSM had consumed alcohol and used drugs at some point in their lives, with marijuana, cocaine, and poppers being the most commonly used substances. Additionally, one of the risk factors for acquiring HIV was not using condoms, especially when under the influence of alcohol and drugs^11^.

Based on the above, it is important to analyze the factors associated with PS consumption in the MSM population of Colombia’s main cities and to describe their consumption profile. This analysis is necessary and relevant for understanding the problem and planning prevention strategies tailored to the actual needs of this population group.

## Materials and Methods

A cross-sectional study was conducted within the framework of a macro research project entitled "Sexual Behavior and HIV Prevalence in Men who have sex with Men in three cities in Colombia: Bogotá, Medellín, and Santiago de Cali, 2019”^12^

In this study, MSM were defined as any biological male who reported having had insertive or receptive manual, oral, genital, or sexual intercourse or practices with other males during the 12 months prior to the study. The sampling started with key participants, known as ‘seeds.’ The sample consisted of 1301 men: 34.43% (448) from Medellín, 33.74% (439) from Bogotá and 31.83% (414) from Cali. Thirteen seeds were used throughout the process, and each participant received three coupons to invite others to the study. This process continued until the sample size was reached in each city. Participants received a primary incentive (a supermarket coupon worth 40,000 COP, approximately 12 USD) and a secondary incentive linked to the successful recruitment of three new participants (cash, 30,000 COP, approximately 9 USD)^13^. Respondent-driving sampling (RDS) was used as it is the most accurate and least biased sample collection technique for hidden populations. This sampling method involves initially recruiting key informants, or ‘seeds,’ who become the first study participants. After participating, they receive three coupons to invite referrals, and this process is repeated until the estimated sample size is achieved. It is important to mention that the seeds, as recruited by the researcher, do not participate in the results, ensuring that the researcher does not interfere in the selection process. This maintains one of the principles of randomness, giving each participant an equal probability of being selected, thus making the sampling probabilistic^13^^, ^^14^.

A survey adapted to the guidelines for repeated behavioral surveys in populations at risk of HIV was used; it was adapted in Colombia by a group of experts from the funding agency^12^^, ^^14^. It consists of 14 sections: social and demographic characteristics, health and access to the general social security system of health, sexual and reproductive history, stable male partner, occasional partners or casual contacts, sexual relations with women, sex work, payment for sex, knowledge and attitudes towards condoms and lubricants, sexually transmitted infections, knowledge, opinions and attitudes towards HIV/AIDS/testing, stigma and discrimination, and social networks.

The dependent variable of the study was PS use in the past 12 months, defined as having used at least one of the following substances: alcohol, marijuana, poppers, cocaine, ecstasy, pipe (cigarette), inhaled glues, or heroin. The independent variables included city, age, socioeconomic status and housing, education level, marital status, occupation, income, tuberculosis diagnosis, gender identity, stable male partner, casual partner, sex work, condom use in the last sexual encounter, discrimination, having someone to help with money, food, or lodging, attending public meeting places, pre-exposure prophylaxis (PrEP), perception of vulnerability to HIV, and HIV status.

To evaluate the association between the participants’ sociodemographic characteristics, risk behaviors, and PS consumption, the chi-square test was used, with a significance level set at <5%. Prevalence ratios (PR) were also estimated with their respective 95% confidence intervals (95% CI). For the multivariate analysis, a model using the Poisson distribution with a log link, and robust estimator was used. Variables included in the model were those that showed significance (p<0.05) in the bivariate analysis. For data analysis, the Nagelkerke R2 value was reported to determine the model’s explanatory power. The final variables that adjusted the model included city, age, educational level, marital status, casual partner, sex work, condom use in the last sexual encounter, discrimination, having someone to help with money, food, or lodging, attending public meeting places (bars, sauna, Internet), and HIV status. Relative and absolute frequency measurements were calculated for each characteristic using RDSAT software. SPSS version 25, licensed by CES University, was used for multivariate analyses. All collected data are freely accessible for consultation on Mendeley Data^15^.

This research was approved by the ethics committee of CES University in session 130 on February 4th, 2019. The study adhered to the Scientific, Technical, and Administrative Standards for Health Research as per Resolution 008430 of October 4th, 1993, issued by the Ministry of Health of Colombia. It was classified as minimal-risk research.

## Results

Of the total study participants, 34.50% (448) were from Medellín, 33.60% (439) from Bogotá, and 31.90% (414) from Cali. About half of the participants were under 25 years old (45.60%). Regarding socioeconomic status, 44.10% belonged to low strata (0, 1, and 2). Approximately four out of ten participants had completed university studies (42.00%), and the majority were single (85.50%). Additionally, 27.80% reported studying and working, while 24.80% indicated having no income.

**Figure 1:**
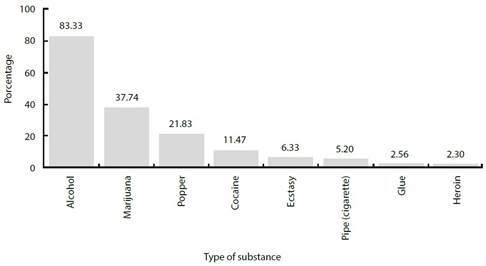
Psychoactive substance consumption, by type of substance, among men who have sex with men, in three Colombian cities, 2019

In terms of sexual orientation, eight out of ten participants identified as homosexual. Additionally, 18.50% had experienced discrimination, 67.40% reported attending public meeting places such as bars, saunas, and Internet venues, and 55.00% reported feeling vulnerable to HIV. The prevalence of PS use in the past year was 87%, with alcohol being the most commonly consumed (83.33%), followed by marijuana (37.74%) and poppers (21.83%) (Figure 1).

The prevalence of PS consumption was significantly higher among individuals under 25 years of age compared to those older than 25 (PR: 1.10; 95% CI 1.06 - 1.15). This result indicates that for every 100 people over 25 who use PS, 110 people under 25 also use them. No relationship was found between economic income or occupation and PS use. However, it was noteworthy that individuals with university-level education had a significantly higher prevalence of PS consumption compared to those without education or with technical or technological studies (p<0.05) (Table 1).

**Table 1:** Psychoactive substance consumption among men who have sex with men, according to sociodemographic characteristics and risk behavior in three Colombian cities, 2019.

PS consumption*					
Variable	Total 1301 %(n)	Yes 1146 %(n)	No 155 %(n)	p-value	cPR (95% CI)**
City				<0.001	
Medellín	34.44 (448)	36.21(415)	21.29(33)		1
Bogotá D.C.	33.74 (439)	32.20(369)	45.16(70)		0.90 (0.86 - 0.95)
Cali	31.82 (414)	31.59(362)	33.55(52)		0.94 (0.90 - 0.98)
Age groups				<0.001	
25 years and older	54.31(705)	51.77(585)	70.43(120)		1
Under 25 years old	45.69(593)	48.23(545)	28.57(48)		1.10 (1.06 - 1.15)
Socioeconomic status				0.13	
Low	44.04(573)	43.37(497)	49.03(76)		1
Middle	53.19(692)	53.85(614)	50.32(78)		1.02 (0.98 - 1.06)
High	2.77 (36)	3.05(35)	0.65(1)		1.12 (1.05 - 1.19)
Educational level				<0.001	
University / Postgraduate	42.16(546)	43.17(487)	35.33(59)		1
None / Preschool / Elementary	4.09(53)	3.46(39)	8.38(14)		0.82 (0.70 - 0.97)
High school	33.44(433)	34.04(384)	29.34(49)		0.99 (0.95 - 1.03)
Technical / Technological	20.31(263)	19.33(218)	26.95(45)		0.92 (0.87 - 0.98)
Marital status				<0.001	
Married / Cohabitating	13.65(177)	13.46(152)	14.88(25)		1
Separated / Divorced / Widowed	0.77(10)	0.44(5)	2.98(5)		0.58 (0.31 - 1.08)
Single	85.58(1110)	86.09(972)	82.14(138)		1.01 (0.95 - 1.08)
Occupation				0.37	
Study and work	27.91 (362)	28.52(322)	23.81(40)		1
Other	13.65 (177)	13.37(151)	15.48(26)		0.95 (0.89 - 1.02)
Work (employee or self-employed)	48.42 (628)	48.45(547)	48.21(81)		0.97 (0.93 - 1.02)
Searching for a job	10.02 (130)	9.65(109)	12.50(21)		0.94 (0.86 - 1.02)
Monthly income				0.93	
More than USD 548	10.18 (132)	10.10(114)	10.71(18)		1
No income	24.90 (323)	25.07(283)	23.81(40)		1.01 (0.93 - 1.09)
Less than a minimum wage	33.54 (435)	33.30(376)	35.12(59)		1.00 (0.92 - 1.08)
Between USD 293 and USD 548	31.38 (407)	31.53(356)	30.36(51)		1.01 (0.93 - 1.09)
Tuberculosis diagnosis				0.27	
No	96.84 (1258)	96.64(1092)	98.22(166)		1
Yes	3.16 (41)	3.36(38)	1.78(3)		1.06 (0.97 - 1.16)
Gender identity				0.95	
Bisexual/Other	20.79 (270)	20.80(235)	20.71(35)		1
Homosexual	79.21 (1029)	79.20(895)	79.21(134)		0.99 (0.94 - 1.05)
Stable male partner				0.11	
Yes	36.19 (469)	35.37(399)	41.67(70)		1
No	63.81 (827)	64.63(729)	58.33(98)		1.03 (0.99 - 1.08)
Casual partner					
No	21.48 (279)	19.20(217)	36.69 (62)	<0.001	1
Yes	78.52 (1020)	80.80(913)	63.31 (107)		1.15 (1.07 - 1.22)
Sex work				<0.001	
No	83.67 (1081)	82.58 (929)	91.02(152)		1
Yes	16.33 (211)	17.42 (196)	8.98(15)		1.08 (1.03 - 1.13)
Condom use in the last sexual encounter				<0.001	
Yes	65.13 (846)	63.89(722)	73.37 (124)		1
No	34.87 (453)	36.11(408)	26.63 (45)		1.05 (1.01 - 1.09)
Discrimination				<0.001	
Yes	17.49 (224)	19.73 (223)	0.66 (1)		1
No	82.51 (1057)	80.27 (907)	99.34 (150)		0.86 (0.83 - 0.88)
Have someone to help with money, food,	or lodging			0.03	
Yes	87.51 (1135)	88.22(996)	82.74 (139)		1
No	12.49 (162)	11.78 (133)	17.26 (29)		0.93 (0.86 - 1.00)
Attend public meeting places (bars, sauna	, internet)			<0.001	
Yes	67.41 (875)	70.35 (795)	47.62 (80)		1
No	32.59 (423)	29.65 (335)	52.38 (88)		0.87 (0.82 - 0.91)
HIV status				<0.001	
Positive	23.74 (312)	21.8(252)	37.97 (60)		1
Negative	76.26 (1002)	78.20(904)	62.03 (98)		1.11 (1.05 - 1.18)

*Values weighted based on population weight PS consumption (Psychoactive substance consumption) ** Crude prevalence ratios (cPR)

The adjusted model explains 17.72% of the variance (Table 2). It was found that MSM who use PS are more likely to be older than 25 years compared to those younger than 25 (aPR: 0.46; 95% CI 0.30-0.71). Similarly, those who reported having one or more occasional partners in the past year are 54% more likely to use PS than those without occasional partners (aPR: 0.44; 95% CI 0.29 - 0.67). This probability also increases among sex workers compared to non-sex workers, with a 53% higher chance of PS use (aPR: 0.47; 95% CI 0.27-0.81). Additionally, those who attend public meeting places (aPR: 3.39; 95% CI 2.34-4.91) and those living with HIV (aPR: 0.65; CI 0.43-0.97) also show an increased probability of PS use.

**Table 2:** Factors associated with psychoactive substance consumption among men who have sex with men, in three Colombian cities, 2019.

Variable	cPR* cPR (95% CI)	p-value	aPR** aPR (95% CI)	p-value
City				
Medellín	1		1	
Bogotá D.C.	0.90 (0.86 - 0.95)	<0.001	2.19(1.36-3.15)	0.001
Cali	0.94 (0.90 - 0.98)	0.011	1.33(0.79-2.22)	0.272
Age				
25 years and older	1		1	
Under 25 years old	1.10 (1.06 - 1.15)	<0.001	0.46(0.30-0.71)	<0.001
Educational level				
University / Postgraduate	1		1	
None / Preschool / Elementary	0.82 (0.70 - 0.97)	0.023	1.6(0.67-3.8)	0.283
High school	0.99 (0.95 - 1.03)	0.516	0.94(0.58-1.15)	0.801
Technical / Technological	0.92 (0.87 - 0.98)	0.019	1.12(0.69-1.81)	0.642
Marital status				
Married / Cohabitating	1		1	
Single	0.58 (0.31 - 1.08)	<0.001	0.89(0.52-1.531)	0.68
Casual partner				
No	1		1	
Yes	1.15 (1.07 - 1.22)	<0.001	0.44(0.29-0.67)	<0.001
Sex work				
No	1			
Yes	1.08 (1.03 - 1.13)	<0.001	0.47(0.27-0.81)	0.007
Condom use in the last sexual encounter				
Yes	1		1	
No	1.05 (1.01 - 1.09)	<0.001	2.10(1.37-3.22)	0.001
Discrimination				
Yes	1		1	
No	0.86 (0.83 - 0.88)	<0.001	1.24(0.68-2.22)	0.476
Have someone to help you if you need money, food, or lodging				
No	1		1	
Yes	0.93 (0.86 - 1.00)	0.03	0.88(0.51-1.54)	0.676
Attend public meeting places (bars, sauna, internet)				
Yes	1		1	
No	0.87 (0.82 - 0.91)	<0.001	3.39(2.34-4.91)	<0.001
HIV status				
Positive	1		1	
Negative	1.11 (1.05 - 1.18)	<0.001	0.65(0.43-0.97)	0.037

*Crude prevalence ratios ** Adjusted prevalence ratios. Adjusted Nagelkerke R2model explains 17.72% of the variance).

## Discussion

The findings of this research indicate a high prevalence of recreational PS consumption, even higher than that found in the general population in Colombia^11^. As in most countries worldwide, marijuana and alcohol were the most commonly used substances^16^^, ^^20^. The high rates of drug use among MSM compared to heterosexual men have been reported in different studies^21^^, ^^22^, exposing this population to a greater risk of different pathologies, such as liver diseases, among others. Excessive alcohol consumption and the use of different types of drugs not only alter cognitive functioning but also significantly affect overall health^22^. For example, cocaine can trigger immediate effects that endanger the user, such as a sudden and excessive increase in heart rate and blood pressure. Long-term consequences of cocaine use include anxiety, insomnia, gastrointestinal problems, and, in severe cases, death due to overdose^23^.

Among the main substances consumed by the MSM participating in this research were alcohol, marijuana, and poppers, a finding consistent with those reported in different contexts. For instance, a study conducted in Spain in 2010 found that among MSM, the most commonly used drugs were cannabis (30.14%), poppers (28.43%), and cocaine (18.7%)^24^. This pattern of substance use may be associated with purchasing power^23^, as well as attendance at high-risk venues, such as bars, saunas, and other social interaction sites. These environments not only increase substance use but also heighten exposure to different risky sexual behaviors^25^.

Some risky sexual behaviors, such as not using a condom in the last sexual encounter (within the past year) and having occasional partners, were associated with substance use. Although this study is not focused on chemsex, it can be inferred that it may be a frequent practice among MSM in the three cities studied. These findings are comparable with other studies. For instance, research conducted in Brazil found that among MSM who use PS, condom use was 30.93%, compared to 53.57% in the group of non-PS users^26^. Similarly, a study in the United Kingdom in 2012 found that polydrug use was associated with a higher prevalence of sex without a condom^22^. In Barcelona, some MSM reported that drug use decreased their perception of risk and directly affected their sexual practices, with cocaine being used as an anesthetic and anal dilator^24^. This pattern of consumption among the MSM population has also been associated with attendance at certain places of sexual contact, such as private parties, saunas, and in general with contexts of barebacking and sexual disinhibition, aligning with the results of this study.

In this study, it was observed that sex work is related to PS consumption. This situation not only increases the physical and mental health risks associated with substance use but also increases the likelihood of risky sexual behaviors among individuals who sell sex. Specifically, it reduces the likelihood of condom use, thereby increasing the vulnerability to HIV among sex workers^27^.

In this sense, understanding the dynamics of the high epidemic infection levels becomes the central issue. According to some authors^22^^, ^^26^, men engaged in sex work may resort to substance use not only due to easier access to them in their line of work but also to desensitize the experiences related to their profession. A study on male sex work in Barcelona found that one of the main problems perceived by sex workers was the consumption of PS as part of their sexual routines. Generally, they reported that substance use was often at the client's request, serving as a sexual stimulant and as a means to endure long working hours^27^.

Another important finding was that MSM who perceived themselves as vulnerable to HIV were more likely to use PS. Similarly, a study conducted in Cali found a higher frequency of HIV diagnosis among those who used drugs (OR: 2.0; 95% CI 1.0 - 4.1) and among those who believed themselves to be at risk (OR: 3.2; CI95% 1.5 - 6.9)^25^. These results indicate a close relationship between PS use among MSM and the risk of contracting HIV, as substance use directly influences decision-making, leading to more frequent adoption of risky sexual behaviors. In Latin America and Colombia, despite the legal allowance for the free development of personality, discrimination against MSM persists, partly due to conservative and religious traditions that foster prejudice. This discrimination can lead to stress, anguish, and risky behaviors, which may result in the consumption of drugs or alcohol as a coping mechanism^28^.

### Limitations

As this is a cross-sectional study, it was not possible to establish temporal sequences or causality. Additionally, since the data are based on self-reports, respondents may have forgotten details, leading to potential inaccuracies in their answers. The RDS method also depends on social networks, which may limit the accuracy of population estimates and affect the results’ generalizability.

## Conclusion

There is a need for substance use prevention programs with a differential approach, considering the interplay between the individual, the substance, and the sociocultural contexts in which consumption occurs. Studies are needed in Colombia to explore the emergence of the chemsex phenomenon and to understand the reasons people engage in these practices. Additionally, STI prevention programs targeting the MSM population should address substance use, as these issues are closely linked. This study allows us to conclude that the factors associated with PS use among MSM increase for those over 25 years old, those who reported having occasional partners in the past year, those who engage in sex work, and those who frequent public or meeting places.
